# 4,4′-Dibromo-2,2′-{ethane-1,2-diylbis[(methyl­imino)­methyl­ene]}diphenol

**DOI:** 10.1107/S1600536811017193

**Published:** 2011-05-14

**Authors:** Augusto Rivera, Jicli José Rojas, Jaime Ríos-Motta, Michal Dušek, Karla Fejfarová

**Affiliations:** a Departamento de Química, Universidad Nacional de Colombia, Colombia; b Institute of Physics ASCR, v.i.i., Na Slovance 2, 182 21 Praha 8, Czech Republic

## Abstract

The asymmetric unit of the title compound, C_18_H_22_Br_2_N_2_O_2_, contains one half-mol­ecule that is related to the other half by a center of inversion located at the mid-point of the central C—C bond. The hy­droxy (phenolic) groups are linked to the N atoms by O—H⋯N hydrogen bonds, which generate *S*(6) rings.

## Related literature

For the synthesis, see: Rivera *et al.* (2010 ▶). For the uses of tetra­hydro­salens in coordination chemistry, see: Atwood (1997 ▶). For a related structure, see: Naza­renko *et al.* (2000 ▶). For reference bond lenghts, see: Allen *et al.* (1987 ▶).
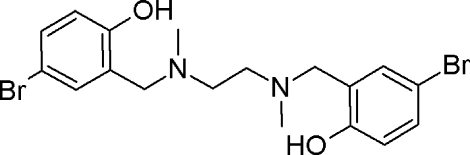


## Experimental

### 

#### Crystal data

C_18_H_22_Br_2_N_2_O_2_
*M* *_r_* = 458.2
Orthorhombic, 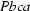

*a* = 15.9282 (3) Å
*b* = 6.1123 (2) Å
*c* = 18.3315 (4) Å
*V* = 1784.72 (8) Å^3^
*Z* = 4
Cu *K*α radiation
μ = 5.87 mm^−1^
*T* = 120 K
0.36 × 0.06 × 0.05 mm

#### Data collection

Oxford Diffraction Xcalibur diffractometer with an Atlas (Gemini ultra Cu) detector
Absorption correction: multi-scan (*CrysAlis PRO*; Oxford Diffraction, 2009 ▶) *T*_min_ = 0.611, *T*_max_ = 1
24526 measured reflections
1591 independent reflections
1482 reflections with *I* > 3σ(*I*)
*R*_int_ = 0.028

#### Refinement

*R*[*F* ^2^ > 2σ(*F* ^2^)] = 0.021
*wR*(*F* ^2^) = 0.075
*S* = 1.52
1591 reflections
112 parameters
H atoms treated by a mixture of independent and constrained refinement
Δρ_max_ = 0.20 e Å^−3^
Δρ_min_ = −0.32 e Å^−3^

### 

Data collection: *CrysAlis PRO* (Oxford Diffraction, 2009 ▶); cell refinement: *CrysAlis PRO*; data reduction: *CrysAlis PRO*; program(s) used to solve structure: *SIR2002* (Burla *et al.*, 2003 ▶); program(s) used to refine structure: *JANA2006* (Petříček *et al.*, 2006 ▶); molecular graphics: *DIAMOND* (Brandenburg & Putz, 2005 ▶); software used to prepare material for publication: *JANA2006*.

## Supplementary Material

Crystal structure: contains datablocks global, I. DOI: 10.1107/S1600536811017193/hb5874sup1.cif

Structure factors: contains datablocks I. DOI: 10.1107/S1600536811017193/hb5874Isup2.hkl

Additional supplementary materials:  crystallographic information; 3D view; checkCIF report

## Figures and Tables

**Table 1:** Hydrogen-bond geometry (Å, °)

*D*—H⋯*A*	*D*—H	H⋯*A*	*D*⋯*A*	*D*—H⋯*A*
O4—H4*o*⋯N2	0.81 (2)	1.86 (2)	2.6051 (19)	154 (2)

**Fig. 1.**
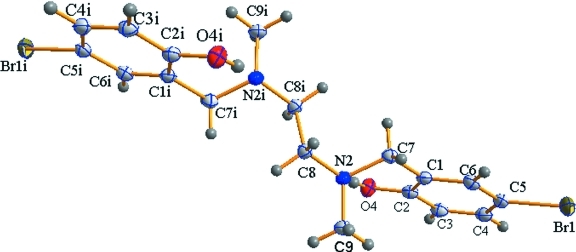
The molecule of the title compound. Displacement ellipsoids are drawn at 50% probability level. Atoms with suffix i are generated by the symmetry operation (1–x, –y, 2–z).

**Fig. 2.**
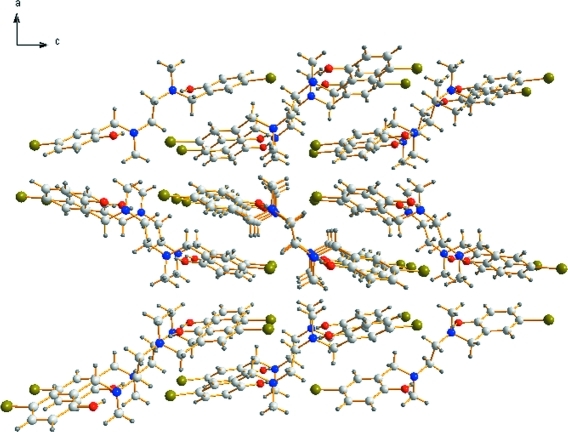
The packing for (I).

## supplementary crystallographic information

### Comment

Recently, we reported the synthesis of a new class of ligands by a ring-opening reduction of bis-1,3-benzoxazines with sodium borohydride (Rivera *et al.*, 2010), and the products of these reactions are referred to as N,*N*'-disubstituted tetrahydro-salens (Atwood, 1997). Here we report the crystal structure of title compound (I). The C(*sp*^3^)—*X* bond distances and angles in (I) are within normal ranges (Allen *et al.*, 1987) and comparable with a related structure (Nazarenko, *et al.*, 2000). The C—N bonds in the N—CH_2_CH_2_—N segment are anti to each other, with a torsion angle of 180°. The observed conformation is stabilized by the short intramolecular hydrogen bonds O—H··· N (Table 1), and these interactions generate S(6) ring motifs.

### Experimental

Sodium borohydride (3.0 mmol, 0.11 g) was added to a solution of 3,3'-ethylene-bis-(3,4-dihydro-6-bromo-2*H*-1,3-benzoxazine) (1 mmol) in ethanol (15 ml), and the mixture was stirred magnetically for 30 min at room temperature. After completion of the reaction, the mixture was poured into ice-cold water, neutralized with ammonium chloride (12 ml), and extracted with CHCl_3_ (3 times 10 cm^3^). The combined extracts were dried over anhydrous Na_2_SO_4_ and evaporated. The solid obtained was purified by recrystallization from ethanol to yield colourless needles of (I).

### Refinement

All hydrogen atoms were discernible in difference Fourier maps and could be refined to reasonable geometry. According to common practice H atoms bonded C atoms were kept in ideal positions with C–H distance 0.96 Å during the refinement. The methyl H atoms were allowed to rotate freely about the adjacent C—C bonds. The hydroxy hydrogen was found in difference Fourier maps and its coordinates were refined freely. The isotropic atomic displacement parameters of hydrogen atoms were evaluated as 1.2×*U*_eq_ of the parent atom.

### Crystal data

**Table:**

C_18_H_22_Br_2_N_2_O_2_	*F*(000) = 920
*M**_r_* = 458.2	*D*_x_ = 1.705 Mg m^−^^3^
Orthorhombic, *P**b**c**a*	Cu *K*α radiation, λ = 1.5418 Å
Hall symbol: -P 2ac 2ab	Cell parameters from 16599 reflections
*a* = 15.9282 (3) Å	θ = 2.8–66.9°
*b* = 6.1123 (2) Å	µ = 5.87 mm^−^^1^
*c* = 18.3315 (4) Å	*T* = 120 K
*V* = 1784.72 (8) Å^3^	Needle, colourless
*Z* = 4	0.36 × 0.06 × 0.05 mm

### Data collection

**Table:**

Oxford Diffraction Xcalibur diffractometer with an Atlas (Gemini ultra Cu) detector	1591 independent reflections
Radiation source: Enhance Ultra (Cu) X-ray Source	1482 reflections with *I* > 3σ(*I*)
mirror	*R*_int_ = 0.028
Detector resolution: 10.3784 pixels mm^-1^	θ_max_ = 67.1°, θ_min_ = 4.8°
Rotation method data acquisition using ω scans	*h* = −18→18
Absorption correction: multi-scan (*CrysAlis PRO*; Oxford Diffraction, 2009)	*k* = −7→7
*T*_min_ = 0.611, *T*_max_ = 1	*l* = −21→21
24526 measured reflections	

### Refinement

**Table:**

Refinement on *F*^2^	41 constraints
*R*[*F*^2^ > 2σ(*F*^2^)] = 0.021	H atoms treated by a mixture of independent and constrained refinement
*wR*(*F*^2^) = 0.075	Weighting scheme based on measured s.u.'s *w* = 1/[σ^2^(*I*) + 0.0016*I*^2^]
*S* = 1.52	(Δ/σ)_max_ = 0.008
1591 reflections	Δρ_max_ = 0.20 e Å^−^^3^
112 parameters	Δρ_min_ = −0.32 e Å^−^^3^
0 restraints	

### Special details

**Table:**

Experimental. CrysAlisPro, Oxford Diffraction (2009), Empirical absorption correction using spherical harmonics, implemented in SCALE3 ABSPACK scaling algorithm.
Refinement. The refinement was carried out against all reflections. The conventional *R*-factor is always based on *F*. The goodness of fit as well as the weighted *R*-factor are based on *F* and *F*^2^ for refinement carried out on *F* and *F*^2^, respectively. The threshold expression is used only for calculating *R*-factors *etc*. and it is not relevant to the choice of reflections for refinement.The program used for refinement, Jana2006, uses the weighting scheme based on the experimental expectations, see _refine_ls_weighting_details, that does not force *S* to be one. Therefore the values of *S* are usually larger than the ones from the *SHELX* program.

### Fractional atomic coordinates and isotropic or equivalent isotropic displacement parameters (Å^2^)

**Table:**

	*x*	*y*	*z*	*U*_iso_*/*U*_eq_	
Br1	0.640851 (13)	0.05571 (4)	0.580303 (11)	0.03125 (11)	
O4	0.61560 (9)	0.4268 (2)	0.88393 (7)	0.0270 (4)	
N2	0.58909 (9)	0.0319 (2)	0.93218 (8)	0.0195 (4)	
C1	0.59115 (9)	0.1285 (3)	0.80150 (9)	0.0202 (4)	
C2	0.62193 (10)	0.3384 (3)	0.81629 (9)	0.0214 (5)	
C3	0.66038 (12)	0.4593 (3)	0.76131 (11)	0.0242 (5)	
C4	0.66647 (10)	0.3757 (3)	0.69103 (9)	0.0251 (5)	
C5	0.63443 (9)	0.1714 (3)	0.67664 (10)	0.0227 (5)	
C6	0.59736 (10)	0.0460 (3)	0.73124 (10)	0.0217 (5)	
C7	0.54916 (10)	−0.0057 (3)	0.86063 (9)	0.0216 (4)	
C8	0.53980 (10)	−0.0646 (3)	0.99219 (9)	0.0218 (5)	
C9	0.67487 (11)	−0.0584 (3)	0.93342 (10)	0.0241 (5)	
H3	0.682855	0.601479	0.77203	0.0291*	
H4	0.692687	0.459508	0.65304	0.0301*	
H6	0.576136	−0.09726	0.720273	0.026*	
H7a	0.490778	0.032417	0.863354	0.0259*	
H7b	0.552676	−0.158184	0.848428	0.0259*	
H8a	0.573654	−0.070797	1.035465	0.0262*	
H8b	0.525375	−0.212723	0.980158	0.0262*	
H9a	0.701148	−0.023134	0.97905	0.0289*	
H9b	0.672436	−0.214496	0.927867	0.0289*	
H9c	0.706892	0.003417	0.894138	0.0289*	
H4o	0.6031 (15)	0.326 (4)	0.9104 (12)	0.0323*	

### Atomic displacement parameters (Å^2^)

**Table:**

	*U*^11^	*U*^22^	*U*^33^	*U*^12^	*U*^13^	*U*^23^
Br1	0.03214 (19)	0.0431 (2)	0.01850 (19)	0.00457 (7)	−0.00120 (6)	−0.00091 (7)
O4	0.0343 (7)	0.0206 (6)	0.0260 (7)	−0.0033 (5)	0.0042 (5)	−0.0036 (5)
N2	0.0153 (7)	0.0236 (8)	0.0195 (7)	−0.0017 (5)	0.0009 (5)	0.0002 (5)
C1	0.0144 (7)	0.0228 (8)	0.0235 (8)	0.0010 (6)	−0.0004 (6)	0.0020 (7)
C2	0.0172 (7)	0.0228 (8)	0.0243 (8)	0.0015 (6)	−0.0011 (6)	0.0006 (7)
C3	0.0210 (8)	0.0226 (9)	0.0291 (10)	−0.0022 (6)	−0.0008 (7)	0.0037 (6)
C4	0.0180 (8)	0.0298 (9)	0.0276 (9)	−0.0002 (7)	−0.0002 (6)	0.0075 (7)
C5	0.0181 (8)	0.0314 (10)	0.0185 (8)	0.0044 (6)	−0.0013 (5)	0.0004 (7)
C6	0.0190 (8)	0.0228 (9)	0.0232 (9)	0.0018 (6)	−0.0032 (6)	0.0008 (6)
C7	0.0194 (8)	0.0244 (8)	0.0210 (8)	−0.0042 (7)	−0.0012 (6)	0.0005 (7)
C8	0.0189 (8)	0.0251 (9)	0.0214 (8)	−0.0012 (6)	0.0012 (6)	0.0036 (6)
C9	0.0163 (9)	0.0289 (10)	0.0271 (8)	0.0019 (6)	0.0000 (7)	0.0004 (6)

### Geometric parameters (Å, °)

**Table:**

Br1—C5	1.9051 (18)	C4—H4	0.96
O4—H4o	0.81 (2)	C5—C6	1.392 (2)
N2—C7	1.476 (2)	C6—H6	0.96
N2—C8	1.475 (2)	C7—H7a	0.96
N2—C9	1.474 (2)	C7—H7b	0.96
C1—C2	1.400 (2)	C8—C8^i^	1.521 (2)
C1—C6	1.387 (2)	C8—H8a	0.96
C1—C7	1.515 (2)	C8—H8b	0.96
C2—C3	1.392 (3)	C9—H9a	0.96
C3—C4	1.389 (3)	C9—H9b	0.96
C3—H3	0.96	C9—H9c	0.96
C4—C5	1.375 (3)		
			
C7—N2—C8	111.79 (13)	N2—C7—C1	111.17 (13)
C7—N2—C9	110.78 (13)	N2—C7—H7a	109.4713
C8—N2—C9	109.40 (13)	N2—C7—H7b	109.4711
C2—C1—C6	119.21 (15)	C1—C7—H7a	109.4712
C2—C1—C7	120.83 (14)	C1—C7—H7b	109.4709
C6—C1—C7	119.93 (15)	H7a—C7—H7b	107.7191
C1—C2—C3	120.04 (16)	N2—C8—C8^i^	112.14 (13)
C2—C3—C4	120.46 (16)	N2—C8—H8a	109.4713
C2—C3—H3	119.771	N2—C8—H8b	109.4716
C4—C3—H3	119.7725	C8^i^—C8—H8a	109.4716
C3—C4—C5	119.09 (16)	C8^i^—C8—H8b	109.4707
C3—C4—H4	120.4567	H8a—C8—H8b	106.6664
C5—C4—H4	120.4562	N2—C9—H9a	109.4705
Br1—C5—C4	119.68 (13)	N2—C9—H9b	109.471
Br1—C5—C6	119.00 (14)	N2—C9—H9c	109.4718
C4—C5—C6	121.31 (16)	H9a—C9—H9b	109.4713
C1—C6—C5	119.85 (16)	H9a—C9—H9c	109.4714
C1—C6—H6	120.0729	H9b—C9—H9c	109.4713
C5—C6—H6	120.073		
			
N2—C8—C8^i^—N2^i^	180		

Symmetry codes: (i) −*x*+1, −*y*, −*z*+2.

### Hydrogen-bond geometry (Å, °)

**Table:**

*D*—H···*A*	*D*—H	H···*A*	*D*···*A*	*D*—H···*A*
O4—H4o···N2	0.81 (2)	1.86 (2)	2.6051 (19)	154 (2)
